# Care of the Deciduous Teeth

**Published:** 1890-04

**Authors:** F. S. Maxwell

**Affiliations:** Steubenville, Ohio.


ARTICLE IV.

### CARE OF THE DECIDUOUS TEETH.

BY F. S. MAXWELL, D. D. S., STEUBENVILLE, OHIO.

[Read at the Ohio Valley Dental Society Meeting, Wheeling, W. Va., Jan. 6, 1890,]

A subject of as grave importance as this, one should take pride in presenting to a dental society for its discussion.

We all know how much good can be obtained by a due consideration of the subject, and for this purpose I

write, hoping it may provoke a general awakening of interest in the right direction.

It must be surprising to all that so little has been written concerning the care of the temporary teeth, inasmuch that we all agree, or should agree, as to their importance. We know that it has only been of late years that they have received any consideration relative to their preservation, but why they have escaped the dentist's care so long remains a mystery.

Perhaps the deciduous teeth of the children of to-day are not as free from decay and disease as they formerly were, and I firmly believe it, and for that reason have never received that proper care heretofore that they are now receiving; however, there is a large field for the care of them alone, at the present time, in which field we should all embark to a considerable extent.

To begin with: a prospective mother should be taught her duty in regard to the future welfare of her offspring. She should be taught of what her system is composed, what effect it has upon her unborn child, what that child derives from her, and what she should make provision for by her diet. Lime, phosphorus, potash, silex, etc., of which the teeth are composed, should be taken in suitable quantities, both for the preservation of her own and her child's teeth; and when I say they should be taken in suitable quantities, I mean, by the taking of food containing these important substances, such as meat, milk, eggs, and vegetables, necessary to produce strong flesh and teeth.

What kind of teeth can you expect in a child in which it and its mother exist on starches and sugars alone? A *little* sugar will do no harm, and it must be pure, but a little does not go far toward forming strong enamel, and they would be better without it altogether. We have all noticed in most mothers' mouths, after child-birth, that pearlish white line or groove, at the gum margin, that could have been prevented to a considerable extent by the free use of lime-water during gestation.

They should be taught that the germs of the child's teeth begin to form as early as the fifth or sixth week of fœtal life, that the teeth become almost solidified before its birth, and that during lactation the diet should be as equally important a factor in the production of strong and beautiful teeth. What delights a mother more than the appearance of her baby's first tooth, and even the succeeding ones, so that if she has followed our advice, her profit is sufficient pay for the trouble taken. Good food means good blood, and all nutritious food taken up by the blood, being assimilated, shows the desired result in the human economy. Too much stress, therefore, cannot be placed upon this part of the care of the deciduous teeth.

We hear and read of plenty of methods of preventing and arresting decay, filling teeth, etc., but for the most part it is for teeth that have been erupted, and but little new can be said in that direction; a little, however, is a step forward.

The first and utmost thing is to impress the importance of cleanliness. A child, ever so small, can be taught the usefulness of a small, soft tooth-brush. The temporary teeth, to them, are as important as the permanent to an adult. Much pain can be saved with but little effort, by the proper use of the brush, and an occasional rinsing of the mouth with lime-water. When they have grown a little older give them a small quill tooth-pick to use, and you will soon see the habit becoming a permanent one.

The deciduous teeth should be filled as soon as decay makes its appearance. But what should they be filled with? Much depends upon circumstances. A child enters your office with the terrible dread of being hurt—some friend has told her "it would almost kill her"—generally, for the first time, accompanied by its mother; I would frequently prefer the mother to remain at home. It enters the chair with a feeling of great fear and hesitancy, and "poor little innocents" look upon the operation, whatever it may be, as a case of life or death. Assure them that you are not near as dreadful a man as you have been pictured, by an explanation of the different instruments brought before them: give them a ride in your pedal-levered chair, a time or two by way of amusement; it takes time, but time is money. Never use the engine for the first time, they have heard of that. Select a sharp, spoon-shaped excavator—the decay is generally of a spongy or leathery texture, and this is the most suitable instrument for removing it, and rose-head burr for smoothing the edges. If the cavity be simple, it will soon be ready for the insertion of an amalgam filling, and the child will feel grateful, and will have satisfied its curiosity.

Do not fill more than one cavity at a sitting, they prefer to come oftener and for a short period. But, suppose this child has been crying for several nights with toothache, an exposed or irritated pulp, and seeks your office for relief! What will you do? Remove the loose decay. Place a small pledget of cotton, saturated with oil of cloves, and source with cotton saturated with gum sondarach. If you have been successful in treatment, in a day or two cap with oil of cloves and oxide of zinc and over that place a thinly mixed phosphate filling, because of its being the easiest to insert without producing pressure. On the other side of the mouth you find its companion, the child says it formerly ached, but does not now give any trouble.

You most likely discover an abscess at the end of a root—dead, or devitalized teeth are prone to abscess. What will you do with it? Will you fill? Certainly. Cleanse the cavity of carious dentine and foreign matter, for the chances are it is alive with "germs," exposing the root canals. Wash out with peroxide of hydrogen until no effervescence is noticed or odor perceptible. Place oil of eucalyptus and iodoform in the canal, or canals, and allow it to remain for three or four days without changing. More harm is done by too much treatment than too little, and if the tooth has been comfortable during that time, fill at once with chlora percha, or gutta percha, if the canal is large enough, preceded by the chlora percha, using care not to force it through the naturally large foramen, and cover with whatever desired. In a deciduous tooth, more care should be taken not to force a foreign substance through the foramen, than is necessary in the permanent, for obvious reasons. The eucalyptus oil saturates the root canals and dental tubuli by virtue of its diffusible properties, and enough remains in the canals to force with chlora percha to the ends of the roots, drying up the fistulous opening and preventing, materially, subsequent trouble.

You notice that I use the essential oils in the treatment of the deciduous teeth. I believe they are the best medicines brought to our notice for that purpose. Suppose capping will not *save*, in your judgment, how will you destroy the pulp? Strangle it with two or three applications of pure carbolic acid. I have used arsenic to destroy but prefer not to in a majority of cases. Remove the pulp with small barbed broaches and fill as before.

There is no trouble, gentlemen, in succeeding with children if we but approach them properly. Treat them tenderly, be patient, delicate in touch, sympathetic, apparently—gain their esteem by kind words, and your path is made much clearer. There is nothing more satisfactory to the mother, child, or yourself, than the successful care and treatment of the child's teeth. Be thorough in your work, earn their confidence by your sound advice and good judgment, and you will be amply repaid by the good results shown and by their subsequent patronage.—*Ohio Journal of Dental Science.*

